# Reconceptualizing anhedonia: novel perspectives on balancing the pleasure networks in the human brain

**DOI:** 10.3389/fnbeh.2015.00049

**Published:** 2015-03-11

**Authors:** Kristine Rømer Thomsen, Peter C. Whybrow, Morten L. Kringelbach

**Affiliations:** ^1^Center of Functionally Integrative Neuroscience (CFIN), University of AarhusAarhus, Denmark; ^2^Department of Psychiatry, Warneford Hospital, University of OxfordOxford, UK; ^3^Centre for Alcohol and Drug Research, School of Business and Social Sciences, University of AarhusAarhus, Denmark; ^4^Semel Institute for Neuroscience and Human Behavior, University of CaliforniaLos Angeles, LA, USA

**Keywords:** wanting, liking, learning, dopamine, opioids, orbitofrontal cortex, depression, schizophrenia

## Abstract

Anhedonia, the lack of pleasure, has been shown to be a critical feature of a range of psychiatric disorders. Yet, it is currently measured primarily through subjective self-reports and as such has been difficult to submit to rigorous scientific analysis. New insights from affective neuroscience hold considerable promise in improving our understanding of anhedonia and for providing useful objective behavioral measures to complement traditional self-report measures, potentially leading to better diagnoses and novel treatments. Here, we review the state-of-the-art of hedonia research and specifically the established mechanisms of wanting, liking, and learning. Based on this framework we propose to conceptualize anhedonia as impairments in some or all of these processes, thereby departing from the longstanding view of anhedonia as solely reduced subjective experience of pleasure. We discuss how deficits in each of the reward components can lead to different expressions, or subtypes, of anhedonia affording novel ways of measurement. Specifically, we review evidence suggesting that patients suffering from depression and schizophrenia show impairments in wanting and learning, while some aspects of conscious liking seem surprisingly intact. Furthermore, the evidence suggests that anhedonia is heterogeneous across psychiatric disorders, depending on which parts of the pleasure networks are most affected. This in turn has implications for diagnosis and treatment of anhedonia.

## Introduction

Pleasure has been proposed to be evolution’s boldest trick allowing species and organisms to seek fundamental, or primary, rewards ensuring survival and procreation in both individuals and species (Kringelbach, 2005; Kringelbach and Berridge, 2009). In contrast, anhedonia is the missing or severe reduction in the ability to fulfil this essential survival function and as such would appear highly evolutionary maladaptive. Yet, anhedonia persists in the general population over shorter and longer time-scales as a key feature of many (if not all) psychiatric disorders, including mood-, addictive-, and eating disorders (Whybrow, 1998). Psychiatric disorders impose a massive societal burden with e.g., major depressive disorder (subsequently referred to as *depression*) having an estimated lifetime prevalence of at least 15% (Kessler et al., 2012). The diagnosis of depression requires that either symptoms of anhedonia or depressed mood are present and is predicted to become the leading cause of disability by the year 2030 (WHO, 2008).

Unfortunately, the growing appreciation of the important role of anhedonia in major psychiatric and neurological disorders has not been matched by a comparable understanding of the underlying neurobiology, and as a consequence treatment options are often limited and mostly unsatisfactory. As an example the evidence suggests that the presence of anhedonia is a predictor of poor treatment response in depression (Spijker et al., 2001) and of relapse in addiction (Koob and Le Moal, 2001; Volkow et al., 2002).

While there are numerous scientific accounts of the neurobiology of disorders such as depression and schizophrenia, few studies have looked specifically at the presence and severity of anhedonia. Since disorders like depression and schizophrenia are characterized by a number of symptoms, findings from these studies are not necessarily related to anhedonia, which has often also not been measured behaviorally (but rather using self-report measures). Recently there has been increasing interest in elucidating the neurobiology of specific psychological behaviors or symptoms, such as anhedonia, rather than disorders *per se* (Hyman and Fenton, 2003; Insel et al., 2010; Der-Avakian and Markou, 2012). The idea is that behavioral processes (or symptoms), such as hallucinations, are more likely than diagnostic categories (such as schizophrenia) to be linked to specific biological components.

Overall, the growing appreciation of the role of anhedonia across psychiatric disorders has not been matched by scientific accounts of the anatomy of anhedonia, and the generally accepted conceptual understanding of anhedonia has been largely unaltered since Ribot first defined it over a century ago as the “inability to experience pleasure” (Ribot, 1896; Snaith, 1992). Recently, however, there has been some progress, summarized in various recent reviews (Gorwood, 2008; Treadway and Zald, 2011; Der-Avakian and Markou, 2012) offering valuable insight on the underlying neurobiology, but with divergent conceptual understandings of anhedonia. While some authors argue in favor of preserving the original definition of anhedonia as reduced subjective experience of pleasure (Der-Avakian and Markou, 2012), other authors make a strong case for distinguishing between deficits in motivation and consummation in anhedonia (Treadway and Zald, 2011).

**Figure 1:**
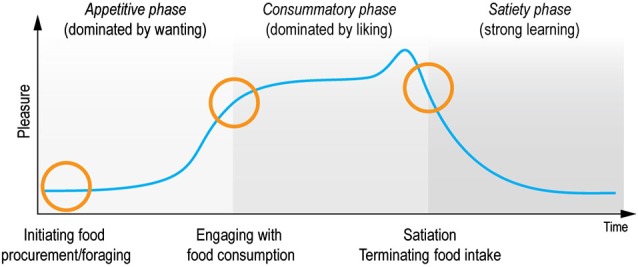
**Pleasure cycle**. The brain needs to optimize resource allocation for survival and individuals are limited in the number of concurrent behaviors. Survival depends on the engagement with rewards and typically follows a cyclical time course common to many everyday moments of positive affect. Within this cycle rewards act as motivational magnets to initiate, sustain and switch state. The cyclical processing of rewards has classically been proposed to be associated with appetitive, consummatory and satiety phases (Sherrington, 1906; Craig, 1918). Research has demonstrated that this processing is supported by multiple brain networks and processes, which crucially involves liking (the core reactions to hedonic impact), wanting (motivational processing of incentive salience), and learning (typically Pavlovian or instrumental associations and cognitive representations) (Berridge and Kringelbach, 2013). These components wax and wane during the pleasure cycle and can co-occur at any time. Importantly, however, wanting processing tends to dominate the appetitive phase, while liking processing dominates the consummatory phase. In contrast, learning can happen throughout the cycle. Here we propose that anhedonia can be conceptualized as specific deficits within this pleasure cycle. Note that a very few rewards might possibly lack a satiety phase (suggested candidates for brief or missing satiety phase have included money, some abstract rewards and some drug and brain stimulation rewards that activate dopamine systems rather directly).

In contrast, the scientific study of hedonia (derived from the ancient Greek word for pleasure: *hedone* from the sweet taste of honey, *hedus*) has undergone substantial progress over the last twenty years. In particular, hedonia research has led to the important discovery, that reward consists of multiple sub-components and processes of wanting, liking and learning that relate to the *appetitive, consummatory and satiety phases* of the pleasure cycle (Robinson and Berridge, 1993, 2003; Berridge and Kringelbach, 2008). The processing of rewards during the pleasure cycle allows individuals to optimize resource allocation for survival (see Figure 1). In this review we use the terms pleasure networks and pleasure system for the brain networks subserving reward processes to underline the importance of pleasure in promoting survival.

Consequently, we show how anhedonia can usefully be conceived as arising from problems with each of these components (wanting, liking, learning) rather than solely being defined as subjective affective *experience of pleasure* as per Ribot’s original proposal. Related to this, we argue that anhedonia can occur on both conscious and unconscious levels, which limits the use of traditional self-report measures (see Box 1). Instead, our reconceptualization allows for the introduction of more objective, scientific measurements of the subcomponents of anhedonia and may in time facilitate the development of more precise diagnoses and perhaps even novel treatments. Thus, anhedonia may have different causes, and effects on subsequent behavior, and these causes and effects can only be examined through more sophisticated methods than self-report.

Box 1 **Anhedonia questionnaires.**

Anhedonia has traditionally been measured with self-report questionnaires. While these can give an indication of the subjective experience of anhedonia, there is evidence from the scientific literature that individuals are not always very good at introspecting their emotional states consisting of both conscious and unconscious components (Kringelbach, 2012). Still these questionnaires have been applied in the diagnosis and study of psychiatric disorders, and offer useful information on the explicit components of anhedonia.

The Chapman Physical and Social Anhedonia Scale (PAS), and its revised version (R-PAS), were developed to measure long-standing, as opposed to transient, anhedonia. Hence, participants are instructed to “describe yourself as you have been during most of your adult life” (Chapman et al., 1976). The scale consists of 61-items (in a true-false format) and measures several domains of pleasure experience, including interest in activities and hobbies, sensory experiences, pastimes, social interaction and food/drink. Psychometrically there has been some disagreement regarding the scale’s construct validity (Germans and Kring, 2000) and discriminant validity (Leventhal et al., 2006). Further, the design of the scale might limit its application in research and clinical settings. With its 61 items it is relatively time consuming, and the content has been criticized for being out-dated (Horan et al., 2006).

The Fawcett-Clark Pleasure Scale (FCPS; Fawcett et al., 1983) is a 36-item questionnaire where participants are asked to rate imagined reactions to pleasurable situations (e.g., “You sit watching a beautiful sunset in an isolated, untouched part of the world”) using a 5-point Likert scale (from “No pleasure at all” to “Extreme and lasting pleasure”). The scale measures several domains of anhedonia, including social activities, sensory experiences, and sense of mastery of difficult tasks; however, none of the domains tap into the incentive salience of reward. Participants are asked to respond based on their current state, thereby measuring anhedonia as a transient state, which makes the scale suitable for evaluation of treatment effects in clinical populations. The psychometric properties of this scale have not been extensively studied, but look promising (Clark et al., 1984; D’haenen, 1996; Leventhal et al., 2006).

The Snaith-Hamilton Pleasure Scale (SHAPS; Snaith et al., 1995) is a brief 14-item questionnaire that assess hedonic tone, or its absence, anhedonia. Participants are instructed to agree or disagree to statements of hedonic response in pleasurable situations (e.g., “I would enjoy my favorite television or radio program”). The scale covers four domains of hedonic experience: interest/pastimes, social interaction, sensory experience, and food/drink, and participants are instructed to respond based on their ability to experience pleasure in the last few days (Snaith et al., 1995). The scale has shown good overall psychometric properties in clinical and non-clinical samples, both in terms of convergent and discriminant validity (Snaith et al., 1995; Gilbert et al., 2002; Leventhal et al., 2006; Franken et al., 2007). The scale is easily applied in clinical and research settings, but only taps into the hedonic impact of reward.

All three questionnaires are routinely used in clinical populations. Because the R-PAS was designed to measure anhedonia as a trait-like characteristic, the scale is less suitable for evaluation of treatment effects in clinical populations. However, in clinical populations with more chronic forms of anhedonia, such as in schizophrenic patients, this scale is often seen as more suitable than FCPS and SHAPS. On a positive note, the R-PAS does not only include items that tap into the hedonic impact of reward, but also includes items that tap into the incentive salience of a reward (in contrast to FCPS and SHAPS). Hence, items like “The sound of rustling leaves has never much pleased me” assess hedonic reactions to activities, while items such as “I have had very little desire to try new kinds of food” assess *interest* in activities, thereby incorporating aspects of the important component of wanting.

Building on the neuroscientific insights reviewed here, The Michigan Wanting and Liking Questionnaire (MWLQ) was recently developed to specifically measure wanting and liking for use in patient groups, including compulsive Parkinson’s patients (Version for Parkinson’s patients with Dopamine Dysregulation Syndrome). The questionnaire was developed by Berridge et al. and measures wanting and liking of normal pleasures, such as food, and of compulsive behaviors, such as pathological gambling activity. The questionnaire consists of five direct contrast questions (e.g., “Overall, which do you usually like or enjoy more: the pleasant experience of gambling (*individually tailored to compulsion*) while you do it, or the pleasant experience of actually eating a favorite food?”) and 17 scaling questions (e.g., “How much do you usually want to eat a favorite food when you are going to eat it just before the meal begins?”). Due to the recent development of this instrument it has not been subject to large scale psychometric testing.

The Sensitivity To Reinforcement of Addictive and other Primary Rewards (STRAP-R; Goldstein et al., 2010) was developed by Goldstein et al. to assess liking and wanting of expected drug rewards as compared to food and sex during three different situations: (a) current, (b) hypothetical, in general, and (c) under drug influence. Participants are asked to *think* about their favorite food, sexual activity and drug or alcohol without reporting the exact stimulus/activity to the interviewer such that privacy is maintained. For liking participants rated “How pleasant would it be to eat it (food), do it (sex) or use/drink it (drug)”. For wanting participants rated “How much do you want to eat it (food), do it (sex) or use/drink it (drug)”. A 5-point Likert scale is used for all questions ranging from 1 (“somewhat”) to 5 (“extremely”). Similar to The MWLQ, the newly developed STRAP-R has not been subject to psychometric testing.

The Temporal Experience of Pleasure (TEPS; Gard et al., 2006) was developed to measure anticipatory and consummatory (online) experiences of pleasure. It is a brief questionnaire consisting of a 10-item anticipatory and an 8-item consummatory pleasure scale, where participants are asked to rate statements using a 6-point Likert scale (from “very false for me” to “very true to me”), e.g., “When something exciting is coming up in my life, I really look forward to it” (anticipatory); “The sound of crackling wood in the fireplace is very relaxing” (consummatory). Examination of convergent and discriminant validity indicate that the two scales measure distinct and specific constructs. In particular the anticipatory scale is related to reward responsiveness and imagery, while the consummatory is related to openness to divergent experiences, and appreciation of positive stimuli. Due to the recency of this instrument, it has only been subject to limited psychometric testing but interestingly has been cross-validated and extended in a Chinese clinical sample of patients with negative and positive symptoms of schizophrenia (Chan et al., 2010).

In the following we first take a brief look at how anhedonia has been measured historically, and outline some of the clinical observations that led us to the proposed reconceptualization of anhedonia. We then discuss pertinent findings regarding the brain networks supporting the wanting, liking, and learning processes underlying the pleasure cycle. We show how the evidence from behavioral and neuroimaging experiments supports the hypothesis of subtypes of anhedonia that reflect impairments in the ability to *experience*, *pursue* and/or *learn* about reward, and discuss implications for the future diagnosis and treatment of anhedonia. We draw on findings from animal studies, and while we stress the need for translational neuroscience, our main focus is on human studies of anhedonia.

## Anhedonia is heterogeneous across major psychiatric disorders

Traditionally anhedonia has been measured with self-report scales or questionnaires like the Fawcett-Clark Pleasure Scale (FCPS; Fawcett et al., 1983) or the Snaith-Hamilton Pleasure Scale (SHAPS; Snaith et al., 1995; see Box 1), which focus on hedonic responses to pleasurable stimuli. However a number of recent questionnaires allow for a differentiation between reward motivation (wanting) and hedonic impact (liking), such as The Temporal Experience of Pleasure Scale (Gard et al., 2006) and The Sensitivity To Reinforcement of Addictive and other Primary Rewards (Goldstein et al., 2010).

While these questionnaires can give valuable information about the subjective experience of anhedonia, there is compelling evidence from the scientific literature that individuals are not always good at introspecting their emotional states consisting of both conscious and unconscious components (Kringelbach, 2012). Still these questionnaires are applied in the diagnosis and study of psychiatric disorders, and offer useful information on the explicit components of anhedonia.

To date, most of the research on anhedonia has been conducted in patients suffering from schizophrenia (Andreasen and Olsen, 1982; Blanchard et al., 2001; Mason et al., 2004; Gooding et al., 2005; Blanchard and Cohen, 2006) and depression (Loas, 1996; Schrader, 1997; Blanchard et al., 2001; Hasler et al., 2004).

Much of the initial research came from the study of schizophrenia, where anhedonia was described as a core symptom from the beginning of the 20th century (Bleuler, 1911; Kraepelin, 1919) and viewed as a stable trait that was genetically transmitted (Rado, 1956). There is a disagreement in the literature as to the role of anhedonia in schizophrenia with some studies stressing that anhedonia is not present in the majority of patients (Chapman et al., 1976), while others suggest that anhedonia is one of two key features involved in the negative symptom complex (Blanchard and Cohen, 2006). In the DSM-5 anhedonia is not directly part of the diagnostic criteria for schizophrenia, but important aspects are captured in some of the negative symptoms: avolition (inability to initiate and persist in goal-directed activities), and affective flattening (absence or near absence of signs of affective expression) (American Psychiatric Association, 2013).

Today, anhedonia is probably most readily recognized in depression where it is one of two main symptoms required for the diagnosis (along with depressed mood). In the DSM-5 criteria for depression the term “anhedonia” is not used explicitly, but is captured in the main criteria as “decreased interest and pleasure in most activities most of the day (American Psychiatric Association, 2013). As we will see, this definition is probably not the most useful as it conflates two important subcomponents of pleasure (i.e., motivation and hedonic impact). But overall, there is an agreement to the importance of anhedonia in depression, and a growing acceptance of the need to more specifically target this symptom to better understand depression and develop improved treatments (Gorwood, 2008; Treadway and Zald, 2011).

In the present review our main focus is on the role of anhedonia in patients suffering from depression or schizophrenia, where most of the work has been conducted (to date). Although there are clear similarities between these disorders regarding the role of anhedonia, it is important to note some of the crucial differences. In depression, anhedonia can be regarded as a transient state (except perhaps in the very severe cases), which is typically defined as a “significant change from before” in the DSM-5. In contrast, anhedonia would appear to reflect a long-lasting (or pervasive) trait-like characteristic in schizophrenia. This difference is supported by findings from a longitudinal study showing that elevated levels of self-reported anhedonia remained stable in schizophrenic patients, but declined in recovered depressed patients after 1-year-follow-up (Blanchard et al., 2001).

Despite the fact that the majority of anhedonia research has been related to depression and schizophrenia, it is important to note the growing evidence that anhedonia also plays an important role across several other psychiatric- and neurological disorders such as drug addiction (Hatzigiakoumis et al., 2011) and Parkinson’s disease (Loas et al., 2012), albeit in heterogeneous ways.

In fact, one of the main arguments for reconceptualizing anhedonia is the notion that anhedonia is expressed differently across disorders, depending on which parts of the pleasure system are most affected, leading to distinct unbalancing in the brain networks.

**Figure 2:**
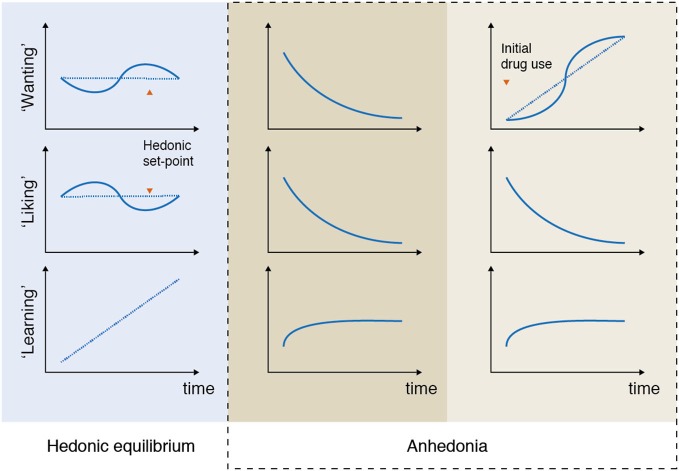
**Anhedonia: examples of unbalancing of pleasure processing in the brain**. In the normal brain, wanting, liking, and learning processes are balanced over time (column 1: hedonic equilibrium). Deficits in some or all of the reward components can lead to various expressions, or subtypes of anhedonia, that are associated with different imbalances of the pleasure system. For example, depressed patients often report a reduced ability to pursue, experience and learn from pleasurable experiences (column 2). This type of imbalance, which is characterized by a progressive decrease in some (or all) of the reward components, is markedly different from the imbalance that characterizes addictive disorders, where “wanting” to take e.g., drugs grows over time independently of “liking” for drugs (column 3). Please note that these illustrations are simplifications of the numerous ways anhedonia can be expressed. For example, according to the available data not all depressed patients lack core “liking” reactions in here-and-now assessments. It is even possible that in some depressed patients core “liking” reactions are retained, but are not cognitively valued as before, which is reflected as reduced liking in self-report inventories rating retrospective and prospective experiences.

For example, in patients suffering from depression anhedonia can be expressed as a reduced ability to experience pleasure *and* a reduced ability to pursue pleasurable activities (Figure 2, column 2). Both of these processes are captured in the DSM-5 criteria where anhedonia is defined as “decreased interest and pleasure in most activities most of the day”, and compromises one of two main symptoms required for the diagnosis (American Psychiatric Association, 2013).

This type of imbalance, which is characterized by a progressive decrease in some (or all) of the reward components, is markedly different from the imbalance that characterizes addictive behavior. One of the core symptoms of drug addiction is the *excess* of wanting for the drug of choice, which in the pathological cases is rarely accompanied by the expected feeling of pleasure (Figure 2, column 3). Although drug “wanting” and drug “liking” are typically strongly linked in the initial phases of drug use, only “wanting” becomes sensitized and consequently increases as the addiction develops (Robinson et al., 2013). The same mechanisms are likely to be at play in behavioral addictions, such as gambling disorder (Rømer Thomsen et al., 2009, 2014).

Generally speaking, anhedonia can be expressed differently across individual patients (sometimes even across time within the same patient as seen most clearly in bipolar disorder, but also in other disorders such as addiction (Nelson et al., 2009)). Importantly, there are also clear differences between the imbalances across psychiatric disorders (as illustrated above), suggesting that anhedonia is a complex psychological process, which consists of several subcomponents, similar to reward (Berridge and Kringelbach, 2008).

## Insights from pleasure research

In the following we outline important findings regarding the underlying brain systems of the subcomponents of reward during the pleasure cycle (Figure 1). Based on this framework we show how deficits in each of these components can lead to different expressions (or subtypes) of anhedonia affording novel ways of measurement, diagnosis, and treatment.

Summarizing a growing body of literature briefly (extensively outlined elsewhere, e.g., Kringelbach and Berridge, 2010; Berridge and Kringelbach, in press), pleasure should be seen within the general framework of evolution as the process by which organisms seek the fundamental rewards ensuring survival and procreation. As such, food and sex are fundamental pleasures, and especially food studies have formed the basis of much hedonia research. In addition, in social species such as humans, social interactions are also fundamental rewards (King-Casas et al., 2005; Kringelbach et al., 2008; Frith and Frith, 2010; Chelnokova et al., 2014). The full repertoire of social pleasures has proven more difficult for experimental investigation and manipulation, yet e.g., the evidence from neuroimaging studies of the role of facial expressions has demonstrated that these pleasures are likely to be as pleasurable as the sensory pleasures (Kringelbach and Rolls, 2003; Rømer Thomsen et al., 2011). Furthermore, humans have the capacity to enjoy higher order rewards, such as musical, artistic, altruistic, and intellectual pleasures. Although the neuroscience of higher order pleasures is still in its relative infancy, there is evidence to suggest that all rewards are translated into a common hedonic currency (Frijda, 2010; Leknes and Tracey, 2010; Vuust and Kringelbach, 2010; Salimpoor et al., 2011).

### Basic pleasure building blocks

Advances in how we define, study, and measure reward have facilitated substantial progress in hedonia research, which form important building blocks in our proposed framework of reconceptualizing anhedonia. In the late 1980s and beginning of the 1990s Kent Berridge and Terry Robinson set the stage for an important turn in hedonia research by proposing to divide reward into the subcomponents of wanting, liking, and learning (Berridge et al., 1989; Berridge and Valenstein, 1991; Robinson and Berridge, 1993).

These conceptualizations have formed the basis of seminal findings. The taxonomy holds that *wanting* is defined as the motivation for, or incentive salience of a reward, while *liking* is the actual pleasure or hedonic impact of a reward. *Learning* is defined as associations, representations, and predictions about future rewards based on past experience, hence representing the time-related perspective of wanting and liking. Each component plays important roles as they wax and wane during the appetitive, consummatory and satiety phases of the cyclical time course of the pleasure cycle (see Figure 1).

**Figure 3:**
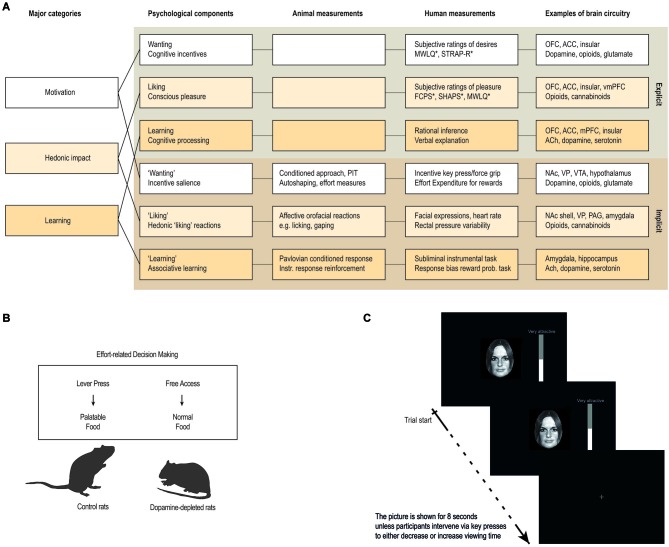
**Measuring anhedonia and hedonia. (A)** Anhedonia is linked to problems with the complex and multifaceted psychological processes involved in hedonia. These include explicit processes of wanting, liking and learning that are consciously experienced and their unconscious counterparts (denoted with quotation marks in the text) that are potentially unconscious i.e., they can operate at a level not always accessible to conscious experience. These components constantly interact and require careful scientific analysis to tease apart. Animal studies have provided measurements or behavioral procedures that are especially sensitive markers of each of the unconscious processes (“wanting”, “liking” and “learning”). Recently, some of these procedures have been successfully translated to human studies, thereby providing more objective behavioral measures to aid subjective self-report measures. In particular, recent developments of behavioral measures of “wanting” and “learning” are promising, while bias-free measures of “liking” reactions in humans have proven more difficult. **(B,C)** Examples of how a measure of “wanting” has been successfully translated from animal to human studies. **(B)** In animal studies, “wanting” can be measured by looking at how willing the animal is to exert effort in exchange for more palatable food rewards, for example by using a choice paradigm devised to look at effort-based decision-making (Salamone et al., 1994, 2007). **(C)** In human studies, “wanting” can be measured similarly, by looking at how much a participant is willing to work for a reward, for example by combining salient stimuli with key-press/force-grip procedures. The first study of this kind used key-presses to operationalize “wanting” as the effort participants exerted to increase or decrease viewing time of images of faces on a screen (Aharon et al., 2001). Abbreviations: OFC: orbitofrontal cortex. ACC: anterior cingulate cortex. vmPFC: ventromedial prefrontal cortex. NAc: nucleus accumbens. PAG: periaqueductal gray. VP: ventral pallidum. VTA: ventral tegmental area. ACh: Acethylcholine. PIT: pavlovian instrumental transfer. *: For questionnaires, see Box 1.

Importantly, these psychological states consist of both unconscious and conscious components (Berridge and Kringelbach, 2008). For example, hedonic impact consists of core *“liking”* reactions (denoted with quotation marks), that are potentially unconscious, and conscious *liking* (without quotation marks), which is the subjective experience of pleasure, capturing the everyday sense of the word as conscious feelings of pleasure or niceness (see Figure 3A).

Similarly, core *“wanting”* reactions are not necessarily conscious and are often triggered by reward-related cues. In contrast *wanting* is the everyday sense of the word as subjective, conscious desires for incentives or declarative goals.

In the same vein, core *“learning”* is the implicit knowledge as well as associative conditioning, such as basic Pavlovian and instrumental associations, while *learning* is the explicit and cognitive associations, representations and predictions about future rewards based on past experience.

This framework has paved the way for a scientific study of pleasure by allowing researchers to quantify, measure, and connect the different components (Berridge and Kringelbach, 2008). This research program has helped to identify the psychological components, measurements and brain circuitry, by extending our knowledge from self-report measures of pleasure in humans with knowledge from behavioral- and physiological procedures, thereby also allowing for a scientific study of unconscious reward components (see Figure 3).

Examples of pleasure-elicited behavioral “liking” reactions are the affective orofacial expressions elicited by the hedonic impact of sweet tastes. These facial “liking” reactions were first described in newborn human infants (Steiner, 1973, 1974; Steiner et al., 2001) and then extended to rodents (Pfaffmann et al., 1977; Grill and Norgren, 1978a,b). Using taste-reactivity paradigms several studies have now shown that sweet tastes elicit positive facial “liking” expressions (i.e., rhythmic licking of lips) in human infants and in rats, whereas bitter tastes elicit facial “disliking” expressions (i.e., gapes.). Since facial “liking” reactions appear to be similar between humans and other mammals (Berridge, 2000; Steiner et al., 2001), findings from animal studies are applicable and useful for our understanding of human pleasure.

Similarly, a useful way to study “wanting” in rodents is to look at food intake and behavior related to obtainment of rewards. Particularly interesting are measures of the effort exerted to obtain pleasurable stimuli, and the ability of reward-related cues to act as motivational magnets. The former can be measured by looking at how eagerly the animal runs for sweet rewards in a runway (Berridge and Valenstein, 1991; Peciña et al., 2003), or how willing the animal is to exert effort in exchange for more palatable food rewards (Salamone et al., 1994, 2007). The latter can be measured by looking at Pavlovian conditioned approach behavior and Pavlovian Instrumental Transfer (PIT; Wyvell and Berridge, 2000, 2001; see Figure 3).

Overall, there is extensive evidence suggesting that the reward system has been conserved across species, and that the same brain structures are involved in affective reactions, whether it is a rat, a monkey, or a human, which makes a strong case for translational research in this area (Ongür and Price, 2000; Berridge, 2003; Berridge and Kringelbach, 2008).

Studies using measures like these yield compelling evidence to support the view that reward is not a unitary process, but is instead a complex process containing several psychological components that correspond to distinguishable, and partly dissociable, neurobiological mechanisms, although the terminology may vary (Berridge and Robinson, 2003; Schultz, 2006; Berridge and Kringelbach, 2008; Leknes and Tracey, 2008). The underlying brain systems of wanting, liking, and learning have been reviewed in detail elsewhere, for a comprehensive review see (Berridge and Kringelbach, in press). Below we briefly review what we know about the underlying brain systems, and particularly, how the components can be dissociated.

### Parsing liking, wanting, and learning

**Figure 4:**
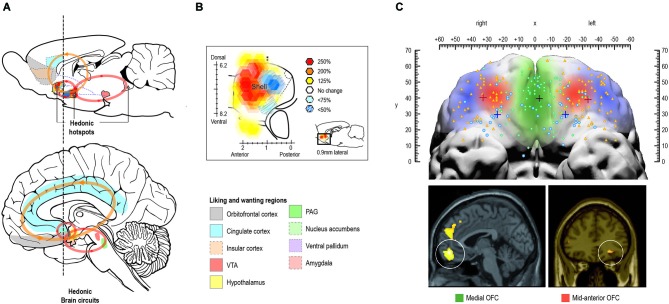
**Pleasure systems in the brain**. The schematic figure shows the brain regions for causing and coding fundamental reward processing in rodents and humans. **(A)** Hedonic causation has been identified in rodents as arising from interlinked subcortical hedonic hotspots, such as in nucleus accumbens and ventral pallidum, where neural activation may increase or decrease “liking” expressions to sweetness, or “wanting” responses to rewards, depending on the specific area of stimulation. Similar pleasure coding and incentive salience networks have also been identified in humans. **(B)** Hedonic hotspots have been found in nucleus accumbens shell and in the ventral pallidum in rodents. **(C)** The cortical localization of pleasure coding may reach an apex in various regions of the human orbitofrontal cortex, which differentiates subjective pleasantness from valence processing aspects of the same stimulus, such as a pleasant food.

The conscious experience of hedonic impact and the underlying “liking” reactions are at the heart of pleasure and is what we intuitively associate with pleasure. Several regions have been found to code for the hedonic impact of reward in the human brain, including cortical regions such as orbitofrontal-, cingulate-, and insular cortex, and subcortical regions such as nucleus accumbens, ventral pallidum, amygdala, and brainstem ventral tegmental area and periaqueductal gray (Kringelbach, 2005; Kringelbach and Berridge, 2009; see Figure 4A).

In the rodent brain, so-called hedonic “hotspots” have been identified, i.e., areas where direct stimulation with microinjections of e.g., opioid agonists can cause or amplify “liking” reactions (Figure 4B). These hotspots have primarily been found in forebrain structures such as nucleus accumbens and ventral pallidum and in the parabrachial nucleus of the brainstem. Stimulation with opioids here, or other signals such as endocannabinoid or orexin, can amplify sensory pleasure by doubling or tripling the normal number of “liking” reactions to sucrose taste (Peciña and Berridge, 2005; Smith and Berridge, 2005; Mahler et al., 2007; Ho and Berridge, 2013).

Only one of the hedonic hotspots in the posterior ventral pallidum appears to be necessary in the sense that damage to it abolishes and replaces “liking” reactions to sweetness with “disliking” (Cromwell and Berridge, 1993). The difficulty of damaging the “liking” generators attests to the robustness of the brain’s capacity for basic hedonic impact processing (Smith et al., 2010) and might offer an explanation as to why hedonic impact can appear to be intact in patients suffering from depression and schizophrenia (at least with here-and-now measures, we will return to this in section Impairments in liking ).

In humans, the mid-anterior orbitofrontal cortex plays a crucial role in the translation of subcortically driven “liking” reactions into our conscious feelings of pleasure and may also be involved in the actual generation of conscious feelings of pleasure (Kringelbach, 2005; Kringelbach and Berridge, 2009; Figure 4C).

The subjective hedonic experience of reward has also been shown to correlate with activity in rostral anterior cingulate cortex (Kable and Glimcher, 2007; Petrovic et al., 2008). Interestingly this activity in rostral anterior cingulate cortex is partially suppressed after naloxone treatment (Petrovic et al., 2008). Equally, the hedonic experience has also been linked to interoceptive mapping (in posterior insula cortex) and “feeling states” (in anterior insula cortex) (e.g., Craig, 2002). Both the rostral anterior cingulate and insula cortices have large concentrations of opioid receptors (e.g., shown in opioid receptor binding PET study by Willoch et al., 2004) and show increased activity following opioid treatment (Petrovic et al., 2002) which could indicate that they may be part of a larger opioid network that has both cortical and subcortical components (Vogt and Sikes, 2000; Fields, 2004).

In contrast the orbitofrontal cortex does not have an equally large opioid-receptor concentration (e.g., Willoch et al., 2004) and unlike rostral anterior cingulate and insular cortices, the orbitofrontal cortex was not found to be active following opioid treatment (Petrovic et al., 2002, 2010). Yet, other positron emission tomography (PET) studies have shown opioid release in the human orbitofrontal cortex linked to placebo and alcohol consumption (Scott et al., 2008; Mitchell et al., 2012). On balance, some of these studies may lend some support to a division where rostral anterior cingulate and insular cortices are more strongly associated with the opioid-dependent liking system (Berridge and Kringelbach, in press), although new tentative findings have identified hedonic hotspots in all of the homologous areas in rodents including the orbitofrontal cortex (Berridge and Kringelbach, in press). This could support the idea that all of these regions are related to the hedonic aspect of reward processing, but also that at least some parts of the orbitofrontal cortex may be more associated with a higher cognitive non-opioid dependent system, possibly the dopamine-dependent wanting system.

Although motivational processes have not traditionally been associated with anhedonia, as per Ribot’s definition, there is increasing evidence that this part of the pleasure cycle is in fact most pertinent in terms of optimizing well-being (Fervaha et al., 2013b; Robinson et al., 2013; Treadway and Zald, 2013). Overall, core “wanting” reactions would appear to be generated in the mesolimbic systems of the brain, in particular those involving dopamine, while the conscious experience of desires and incentives recruits cortical regions, including orbitofrontal-, cingulate-, and insular cortex (see Figure 4).

Mesolimbic dopamine was long considered a pleasure neurotransmitter involved in the hedonic impact of reward (e.g., Wise, 1980), but increasing evidence now suggests that this is not the case. Studies teasing apart “liking” and “wanting” have convincingly shown that specific manipulation of dopamine signaling fails to shift “liking” reactions to pleasure reliably in animals and humans (Berridge and Valenstein, 1991; Peciña et al., 2003; Ward et al., 2012). Instead, evidence points to an important role of dopamine in “wanting” processes. For example, studies show that elevation of dopamine in rats makes the animal run more eagerly towards sweet rewards and cause increases in food consumption (Berridge and Valenstein, 1991; Peciña et al., 2003) and increases the animal’s willingness to work for food reward (Bardgett et al., 2009), while attenuation or blockade of dopamine has the opposite effect (Cousins and Salamone, 1994; Salamone et al., 2007). Similarly, overexpression of D2 receptors impairs an animal’s willingness to work for a reward, while “liking” reactions are preserved (Ward et al., 2012). Studies using PIT paradigms or progressive ratio schedules also support the notion that dopamine plays a crucial role in the motivational processes of hedonia and anhedonia (Barr and Phillips, 1999; Der-Avakian and Markou, 2010; Venugopalan et al., 2011; Peciña and Berridge, 2013).

Similarly, human studies show that elevated levels of dopamine, induced by amphetamine or L-Dopa, increase ratings of wanting for the drug, but not ratings of liking when actually taking the drug (Leyton et al., 2002, 2007; Liggins et al., 2012; see Figure 2). Notably, amphetamine-induced elevated dopamine has recently been shown to increase willingness to work for rewards, thereby providing evidence that dopamine affects “wanting” in humans using a more objective, behavioral measure (Wardle et al., 2011).

Similar to dopamine, elevation of opioids in rats increases “wanting” reactions. For example, it has recently been shown that dopamine and opioid stimulation of nucleus accumbens similarly amplify cue-triggered “wanting” for reward in a study using a PIT paradigm (Peciña and Berridge, 2013). Importantly, morphine-induced elevated levels of opioids were recently shown to increase willingness to work for a reward in humans (using a behavioral measure), while naltreoxone-induced decreased levels had the opposite effect (Chelnokova et al., 2014). Notably, the same study provided similar evidence of the role of opioids in reward liking in humans (i.e., stimulation of the opioid system enhanced self-reported liking ratings while the antagonist had the opposite effect), in line with animal studies.

Still, the interactions between the opioid-dependent liking system and dopamine-dependent wanting system are not fully understood at this time. For example, a study has found an increased subjective liking associated with amphetamine treatment—which can be suppressed after naltrexone treatment (Jayaram-Lindström et al., 2004). Equally, evidence is emerging that there is a dynamic interdependency between goal-directed and habitual systems (Wassum et al., 2009). This suggests that increased dopamine activity can also increase opioid activity to rewards, and in general the interactions between these neurotransmitter systems are important to investigate in future.

The evidence suggests that areas that cause “wanting” reactions are more widespread in the brain than areas that cause “liking” reactions. For example, in the nucleus accumbens shell, the hedonic hotspot (where opioid stimulation amplifies “liking” reactions) is only a cubic millimeter in size, while the entire medial shell mediates opioid-stimulated increases in “wanting” (Zhang et al., 2003; Smith et al., 2011; Peciña and Berridge, 2013). This may predispose us more naturally to states of desire than to states of hedonic impact (Robinson et al., 2013).

Taken together, the evidence shows that wanting and liking are partly dissociated in the brain. Although we generally want what we like and vice versa this is not always the case. This is particularly evident in drug addiction, which is characterized by an excess of craving for drugs, which is rarely matched by a comparable positive hedonic impact (Robinson and Berridge, 1993; Robinson et al., 2013). Further, while conscious and unconscious components are usually linked, this is not always the case. For example, a core “liking” reaction can also happen without subjective awareness (Berridge and Winkielman, 2003; Winkielman et al., 2005).

Although it is more challenging to parse “wanting” and “learning” evidence suggests that it is possible to parse learned predictions apart from “wanting” (incentive salience) (Berridge et al., 2009; Smith et al., 2011). One line of evidence comes from neural coding studies of “wanting”, particularly after dopamine-elevated brain activity (by amphetamine or prior sensitization). While dopamine elevation seems to enhance neural firing to signals that encode maximal incentive salience, it does not enhance neural signals that code maximal prediction (Tindell et al., 2005).

Another line of evidence comes from studies where “wanting” of a conditioned stimulus is reversed, while the learned prediction remains the same. For example, a cue predicting saltiness would normally not be “wanted”, but if a salt appetite is induced, the cue will suddenly turn into a “wanted” cue (Robinson and Berridge, 2013). This change in motivation is not dependent on new learning or changes in learned predictions.

Overall, these findings indicate that “wanting” and “learning” have distinct psychological identities and distinguishable neural substrates—although more studies are needed before we can determine how these psychological states are parsed within the brain.

## Reconceptualizing anhedonia

These new insights from the study of pleasure in humans and other animals open up the possibility of reconceptualizing anhedonia to reflect the heterogeneous and complex nature of reward processing. Based on the framework developed by Berridge and Robinson we propose to conceptualize anhedonia as potential impairments in wanting, liking and learning components, which can lead to different expressions, or subtypes of anhedonia, depending on which parts of the pleasure networks are most affected. In the normal brain, wanting, liking and learning processes are balanced over the pleasure cycle and over longer time scales, but impairments in each of the components can lead to a breakdown of this balance (see Figure 2). This breakdown can be temporary (e.g., as seen in depression) or longer lasting (as seen e.g., in schizophrenia) and can manifest itself in different ways to self-report measures (see Box 1; Figure 3).

In the following we review the evidence suggesting that anhedonia can be expressed as impairments in the ability to experience, pursue, and/or learn from reward, and discuss how these processes can be measured on different levels of analysis that can aid traditional self-report measures. This leads to our proposed reconceptualization of anhedonia and a discussion of how the components of anhedonia are affected across major psychiatric disorders (Figures 2, 3).

### Impairments in liking

In humans the most straightforward way to measure liking is to ask people to self-report using various scales and questionnaires to quantify the experienced pleasure of different stimuli or activities. However, self-report is not always a reliable indicator of the state of the underlying pleasure networks. Studies have shown that what we subjectively report as pleasurable is not always in accordance with our behavior (Aharon et al., 2001; Winkielman et al., 2005; Moeller et al., 2009) and there is evidence that reward affects our behavior, even when we are not consciously aware of it (Winkielman et al., 2005; Pessiglione et al., 2007, 2008; Aarts et al., 2008). Still, these measures are used and provide valuable information on the explicit components of anhedonia.

#### Self-report measures of liking

The literature of changes in hedonic impact processing in patients with psychiatric disorders is highly heterogeneous and has used a variety of self-report measurements (including self-report questionnaires, see Box 1).

A popular way of measuring liking in humans is to assess self-reported hedonic reactivity (i.e., ratings of pleasure) and sensitivity (i.e., identification and threshold) to various pleasant solutions and odors in a here-and-now setting. As such, it resembles the taste-reactivity paradigm, which has been successfully used in animals and newborn babies, but with the important difference that it is based on *self-report*. This paradigm has been used to study reduced liking in depressed patients and shows mixed findings in terms of sensitivity. While some studies show reduced sensitivity to pleasant gustatory and olfactory stimuli (Berlin et al., 1998; Pause et al., 2001; Lombion-Pouthier et al., 2006; Clepce et al., 2010; Negoias et al., 2010), other studies report normal levels of identification and perception thresholds in depressed patients (Scinska et al., 2004, 2008; Swiecicki et al., 2009; Clepce et al., 2010).

Importantly, most studies of depressed patients and non-clinical participants with depressive symptoms report similar, or higher, pleasantness ratings to sweet solutions (Amsterdam et al., 1987; Berlin et al., 1998; Scinska et al., 2004; Swiecicki et al., 2009; Dichter et al., 2010) and various odors (Steiner et al., 1993; Pause et al., 2001; Lombion-Pouthier et al., 2006; Scinska et al., 2008; Swiecicki et al., 2009; Clepce et al., 2010), compared to healthy controls. Similarly, studies of patients suffering from schizophrenia fail to show decreased hedonic reactivity to pleasurable stimuli in comparison to healthy controls (Heerey and Gold, 2007; Barch and Dowd, 2010; Strauss and Gold, 2012). In contrast to this, patients suffering from depression and schizophrenia report reduced enjoyment when asked to rate *prospective, retrospective*, or *hypothetical* experiences (McFarland and Klein, 2009; Watson and Naragon-Gainey, 2010; Strauss and Gold, 2012).

The majority of studies tapping into hedonic reactivity and sensitivity have been done with depressed and schizophrenic patients, while studies looking specifically at anhedonic symptoms are lacking. Part of the conflict between the hypothesis of reduced liking and findings of normal levels in these patients may benefit from a focus on anhedonic symptoms *per se*. For example, an inverse relationship between hedonic responses to sucrose and physical anhedonia scores has been found (Berlin et al., 1998). Similarly, a recent study looking at olfactory hedonics in patients in a depressive state, a remitted state and healthy controls, found no differences in hedonic and intensity estimates between groups. However, during the depressive state, they found a negative relation between anhedonia symptoms and olfactory hedonics, with high scores on the SHAPS being related to low hedonic estimates (Clepce et al., 2010).

Surprisingly few studies have looked at hedonic reactivity and sensitivity in unipolar vs. bipolar patients. Bipolar patients are of particular relevance as their hedonic capacity, or at least their cognitive construal about hedonic experiences, is likely to be affected by changes to their current state (i.e., whether they are in an acute manic or depressive episode).

A recent study looked at hedonic reactivity and sensitivity to pleasant/unpleasant olfactory and gustatory stimuli in unipolar and bipolar patients (Swiecicki et al., 2009). They reported no differences between groups in measures of sensitivity, but bipolar patients, compared to unipolar patients, tended to rate gustatory stimuli as more unpleasant and olfactory stimuli as more pleasant. Unfortunately, the study did not report whether the bipolar patients were in a manic or depressive episode at time of testing.

So far, studies investigating sensory pleasures in anhedonic patients have primarily focused on taste and odor, while other sensory pleasures such as touch remain unexplored. Findings from these studies are potentially highly relevant, but more studies are needed before we can determine if the hedonic capacity across sensory pleasures is attenuated in anhedonia.

#### Physiological measures of liking

It is crucial that self-report measures are combined with more objective measures of “liking” reactions. However, finding bodily markers of emotional feelings and pleasure “liking” in humans is challenging (Steiner et al., 2001), and we are still in need of proper methods. For instance, the orofacial “liking” reactions to sweet and bitter taste, which have formed the basis of seminal findings in pleasure research in rodents and other animals, are not easily transferred to human studies. With time humans learn to carefully control these behavioral reactions, either by inhibiting or imitating them, which limit the use of them as objective markers of pleasure and emotional feelings. Some physiological measures have been used, e.g., showing that people who score high on self-reported measures of anhedonia show hypo-responsiveness on measures of heart rate and facial expression to emotion-eliciting pictures (Ferguson and Katkin, 1996) and scripts (Fiorito and Simons, 1994) and report lower hedonic responses to emotion-eliciting pictures (Ferguson and Katkin, 1996) and scripts (Fiorito and Simons, 1994).

Although physiological measures are more objective in nature, and as such avoid some of the bias inherent in self-report, they are often non-specific in nature and thus difficult to interpret. For instance, with measures such as heart rate, skin conductance response or respiration depth, the inherent non-specificity of these measures means that it is difficult to evaluate whether responses are due to changes in positive or negative affect. Electromyographic (EMG) recordings are effective in detecting emotion-related facial movements, including movements that are not visible to observers (Dimberg, 1982, 1990). Studies have revealed that we react with distinct facial EMG in response to emotional facial expressions (partly reflecting a tendency to mimic the facial stimuli) (Dimberg and Thunberg, 1998), and these reactions have been demonstrated even in conditions where participants are unconsciously exposed to facial stimuli (Dimberg et al., 2000). Although it is unlikely that all changes in facial musculature are emotion-related, recordings of EMG reactions could provide a promising mean of investigating deficits in “liking” reactions to facial stimuli. EMG reactions have been related to e.g., empathy (Dimberg et al., 2011), but more work is needed to confirm that these facial reactions are faithful indicators of reward “liking”.

Other physiological measures, which may be more bias-free and straightforward to interpret, are measures specific to sexual pleasures. For example, Georgiadis et al. measured rectal pressure variability in combination with self-reported perceived level of sexual arousal to distinguish between female sexual arousal, simulation of and real orgasms (Georgiadis et al., 2006). To our knowledge, this type of measure has not been used in patients with self-reported anhedonia symptoms, but represents a promising tool to help elucidate impairments relating to sexual activity.

#### Neuroimaging measures of liking

Several neuroimaging studies have investigated the neural correlates of anhedonia in terms of reduced liking, typically by using self-report measures of pleasure liking and/or emotional visual stimuli (e.g., pictures of happy and sad faces), or by looking at neural responses to receiving a reward. Related to this, recent studies have used the Monetary Incentive Delay (MID) task, which distinguishes between reward anticipation and consummation, similar to wanting and liking (Knutson et al., 2000).

In studies of depressed patients (or participants with elevated symptoms of self-reported anhedonia) findings consistently show a positive correlation between levels of anhedonia and ventromedial prefrontal cortex (VMPFC) activity (extending to orbitofrontal and anterior cingulate cortices) in response to positive/pleasant stimuli (Kumari et al., 2003; Mitterschiffthaler et al., 2003; Keedwell et al., 2005; Epstein et al., 2006). Further, findings show a negative association between anhedonia severity and activity in subcortical regions, particularly in ventral striatum, in response to positive/pleasant stimuli (Limousin et al., 1995; Dunn et al., 2002; Keedwell et al., 2005; Surguladze et al., 2005; Epstein et al., 2006; Wacker et al., 2009). Overall, studies of depressed patients (and not anhedonia *per se*) have revealed diminished activity in striatum, particularly ventral striatum, in response to receipt of reward (McCabe et al., 2009, 2010; Pizzagalli et al., 2009; Smoski et al., 2009).

In patients suffering from schizophrenia there is also evidence of blunted ventral striatum responses to reward receipt, although findings are more mixed (possibly reflecting the fact that this patient group is more heterogeneous). In general, however, studies have reported an association between reduced striatal responses to reward receipt and increased negative or depressive symptoms (Waltz et al., 2009, 2010; Simon et al., 2010).

These findings lend support to the hypothesis that the anhedonia seen in patients can be characterized by specific changes to the pleasure networks through dual changes in activity in ventral striatum (including the nucleus accumbens) and in the prefrontal cortex (including the VMPFC and orbitofrontal cortex) (Gorwood, 2008; Willner et al., 2013). Such ideas would fit well with findings from Berridge et al. who have shown that stimulation with opioids in the nucleus accumbens (shell) and in the ventral pallidum increases “liking”, as illustrated by the so-called hedonic hotspots (Peciña and Berridge, 2005; Smith and Berridge, 2007). In addition, the recent study by Chelnokova et al. points to a similar role of opioids in human liking (Chelnokova et al., 2014), although human hedonic hotspots have not been demonstrated to date.

#### Summary

Overall, there are conflicting findings in the literature and at the moment the evidence does not support the simple hypothesis that anhedonia is always accompanied by reduced liking ratings and associated “liking” reactions to pleasurable stimuli. Taste-reactivity studies measuring self-reported liking in here-and-now settings show normal levels of enjoyment in patients suffering from depression and schizophrenia. In contrast, studies measuring prospective, retrospective and hypothetical experiences of pleasure find reduced levels of liking in these patients.

It is important to stress that the reported finding that here-and-now measures of liking are surprisingly intact in depressed and schizophrenic patients is based only on *self-report*. Future studies applying valid behavioral or physiological measures may inform us differently, and are needed before we can make conclusive statements.

Findings from imaging studies have revealed blunted responses to rewards in a network of structures including subcortical regions, which could point to a reduced “liking” reaction, but these measures need to be accompanied by valid behavioral measures. One of the great challenges is to find valid measures and bodily markers of core “liking” processes in humans that can be applied in neuroscience.

### Impairments in wanting

Similar to liking, a straightforward way to measure wanting is to ask people about their desires. Further, a number of promising behavioral tasks have recently been developed that measure “wanting” processes, primarily by looking at how much participants are willing to work for a reward. This translation of measures from the animal literature, where effort-based measures of behavior have long been used to study motivational processes, is promising, and may allow us to investigate “wanting” processes that are outside our conscious awareness and control (see Figure 3). At the same time, proper use of these methods is crucial for valid interpretation of the data.

#### Behavioral measures of wanting

Aharon et al. developed one of the first behavioral measures of “wanting” for use in humans (Aharon et al., 2001). In their key-press task, “wanting” was operationalized as the amount of work participants performed in order to change the relative duration they viewed images of average and beautiful faces. The study found a difference between self-reported liking ratings and effort, with heterosexual males rating beautiful female and male faces as equally attractive, but using more effort to keep the female faces on the screen. We and other groups have used similar measures of effort, and e.g., found support for a dissociation of conscious appraisal (liking) and behavioral responsivity (“wanting”) to infant faces (Parsons et al., 2011).

Importantly this type of measure has now also been used in patients. In a study of cocaine addiction, Moeller et al., showed that cocaine addicted used more effort to view cocaine-related pictures compared to control participants. Furthermore, there was a discrepancy between self-reported ratings and behavior: while cocaine addicted rated pictures of pleasant scenes as more pleasant than cocaine-related pictures, they did not show this preference in the behavioral choice task (Moeller et al., 2009). This dissociation, or impaired insight, is in line with previous findings of a disconnection between subjective and objective markers of behavior in drug addiction (Goldstein et al., 2007, 2008, 2009; Hester et al., 2007). Impaired insight and self-control is an important feature of drug and behavioral addiction (Goldstein et al., 2009; Changeux and Lou, 2011; Rømer Thomsen et al., 2013; Moeller and Goldstein, 2014; Voon et al., 2014), which underscores the need to compliment self-report ratings with behavioral measures in these patients.

Other groups of researchers have used a related and promising measure of effort by using a handgrip device in combination with subliminal priming paradigms to measure motivational processes outside of our awareness (Pessiglione et al., 2007; Aarts et al., 2008). Aarts et al. showed that subliminally priming of the concept of exertion prepares people to display forceful action, and when these subliminal primes are accompanied with a positive stimulus it motivates people to spend extra effort (Aarts et al., 2008). Pessiglione et al. used a similar set-up to look at unconscious motivation by using an incentive force task that varied the amount and reportability of monetary rewards for which participants exerted physical effort (Pessiglione et al., 2007). In line with Aarts et al., findings showed that even when participants cannot report how much money is at stake, they still deploy more force for higher amounts. This type of effort measure has not been applied to samples of patients with anhedonia yet, but represents a promising way to investigate “wanting” processes that are not necessarily conscious.

Another good example of how animal models of motivation can be translated to human studies comes from Treadway et al. who developed an effort-based decision-making task (the “effort expenditure for rewards task”, EEfRT) (Treadway et al., 2009) based on an animal model (Salamone et al., 1994). In the task reduced “wanting” is operationalized as a decreased willingness to choose greater-effort/greater-reward over less-effort/less-reward options with varying probability. Initially the task was employed in a student sample, where they found an inverse relationship between self-reported anhedonia and willingness to expend effort for rewards. Recently, the task has been employed in relevant patient groups. Compared to controls, patients with subsyndromal depression, first-episode depression or with remitted depression were less willing to expend effort for rewards (Treadway et al., 2012a; Yang et al., 2014). Similarly, two recent studies reported decreased willingness to expend effort for rewards in patients suffering from schizophrenia (Fervaha et al., 2013c; Gold et al., 2013). These findings are promising, however, it shoud be noted that in these tasks, unlike the animal models, the human participants are not working for fundamental rewards but for monetary reward. It is an open question whether abstract rewards such as money are treated in the same way as fundamental rewards, but there is emerging evidence to suggest that there are important differences in the underlying brain processing (Sescousse et al., 2013a,b).

#### Neuroimaging measures of wanting

To our knowledge, no imaging studies have directly investigated changes in “wanting” in a patient group with anhedonia. Although the EEfRT has been applied to relevant groups of patients, findings from imaging studies have not yet been published. Recently, however, the task has been used to investigate the role of dopamine in effort-based decision-making by using PET imaging and dopaminergic manipulation (Wardle et al., 2011; Treadway et al., 2012b). Further, imaging studies using gambling tasks that provide valuable information on reward anticipation (albeit without behavioral measures) have been applied to relevant patients. Lastly, findings from studies measuring wanting in healthy participants are potentially informative of the mechanisms that are impaired in patients with anhedonia.

As reviewed in section Parsing liking, wanting, and learning, mesolimbic dopamine circuitry has consistently been shown to play a crucial role in “wanting” responses in animals. Recently, Wardle et al., provided some of the first evidence that dopamine affects “wanting” similarly in humans, by showing that administration of the dopamine agonist d-amphetamine produces dose-dependent increases in the willingness to work for rewards, as assessed by the EEfRT (Wardle et al., 2011). A subsequent PET-study showed that individual differences in dopamine function in left striatum were positively correlated with willingness to expend greater effort for larger rewards (particularly when probability of reward was low, which is a general finding with this task) (Treadway et al., 2012b).

These findings are in line with findings from functional magnetic resonance imaging (fMRI) studies using gambling tasks to investigate reward anticipation, which have shown diminished responses to anticipation of reward in the ventral striatum in patients suffering from depression (Forbes et al., 2009; Smoski et al., 2009) and schizophrenia (Juckel et al., 2006a,b; Simon et al., 2010; Dowd and Barch, 2012).

Taken together, the data provides strong support for a critical role of striatal dopamine function in effort-related behavior, mirroring findings from animal studies (Salamone et al., 2007; Berridge and Kringelbach, 2008) and psychopharmacological findings in humans (Wardle et al., 2011).

Studies that have applied behavioral measures of “wanting” in healthy controls also highlight the role of subcortical reward areas. Using fMRI Aharon et al. revealed activity changes in parts of the pleasure system, particularly the nucleus accumbens, during passive viewing of beautiful female faces, while a more complex set of subcortical and paralimbic reward regions followed aspects of the key pressing procedure (Aharon et al., 2001). This is in accordance with findings from animal studies consistently showing that “wanting” mechanisms include larger networks in the brain, compared to “liking” mechanisms, which are very localized (Zhang et al., 2003; Smith et al., 2011; Peciña and Berridge, 2013).

The study by Pessiglione et al. showed that even when participants are unable to report how much money is at stake, they still use more effort in terms of force for higher amounts of money. Analysis of corresponding fMRI data revealed that the reported unconscious motivational effect was underpinned by bilateral engagement of the ventral pallidum (Pessiglione et al., 2007). Their findings thus suggest that this region is a key node in the brain circuitry that enables expected rewards to energize behavior without the need of the participants’ awareness.

The reported role of the human ventral pallidum in incentive motivation (“wanting”) accords well with findings from rodents, which have consistently shown that the ventral pallidum is key to goal-directed incentive salience processes (Smith and Berridge, 2005; Tindell et al., 2005; Aldridge and Berridge, 2010). Elevation of dopamine in ventral pallidum appears to specifically enhance neural firing to signals that encode maximal incentive salience in rodents (Tindell et al., 2005). Similar to the nucleus accumbens shell, hedonic “liking” and “wanting” are systematically mapped in a neuroanatomically and neurochemically interactive manner in the ventral pallidum (Smith and Berridge, 2005).

#### Summary

Following the literature in other animals, the wanting or the motivational salience of rewards can now be investigated using behavioral tasks in humans, measuring the amount of work that participants are willing to expend for rewards.

Overall, the available data suggests that deficits in motivational aspects of pleasure play an important role in anhedonia, as evidenced by findings that patients suffering from depression and schizophrenia are less willing to work for a reward, compared to controls. The idea that motivational processes are as important in anhedonia as hedonic impact was proposed already in the 1990s (Willner et al., 1998; Kring, 1999; Germans and Kring, 2000), and following recent successful developments of behavioral tasks that measure motivational aspects of pleasure processing in humans, the idea has gained renewed interest (Treadway and Zald, 2011, 2013; Strauss and Gold, 2012).

Furthermore, there is direct evidence of the role of dopamine and opioids in the regulation of “wanting” processes, and imaging studies of healthy participants mirror findings from animal studies by stressing the role of subcortical reward areas, such as ventral pallidum and nucleus accumbens. However, patient populations have yet to be extensively tested using effort-based measures in combination with neuroimaging, which leaves much scope for a better characterization of the underlying networks involved in the reduced ability to pursue pleasure. The development of effort-based measures of behavior is promising and holds great promise in terms of investigating “wanting” processes that are outside our conscious awareness and control (see Figure 3).

### Impairments in reward learning

A large number of animal studies have looked at the ability to optimize behavior based on past experiences with rewards and punishers using e.g., decision-making tasks. This literature has elucidated some of the fundamental principles of learning involved in reward processing and there is evidence that patients suffering from anhedonia show impaired reward learning.

#### Behavioral measures of reward learning

A number of studies have looked at anhedonia using probabilistic reward tasks that tap into the learning component of reward.

Pizzagalli et al. have used a probabilistic reward task which measures the propensity to modulate behavior based on positive reinforcement history. The task is based on signal-detection theory and was originally developed by Tripp and Alsop (Tripp and Alsop, 1999). In the task, an asymmetrical reinforcer ratio is used (i.e., one stimuli is rewarded more frequently than another) and one of the main outcome measures is the propensity to develop a response bias toward the more rewarding stimulus. In the first study, Pizzagalli et al. showed a different reward learning pattern in participants with low vs. high levels of depressive symptoms. While both groups developed a response bias toward the more rewarding stimulus (i.e., indicative of a functioning “learning” system), the response bias only increased over time (from block 1 to block 3) in the group with low levels of depressive symptoms (Pizzagalli et al., 2005). Subsequent studies of patients showed that clinically depressed patients had problems integrating reinforcement history over time and failed to show a response bias toward the more rewarding cue in the absence of immediate reward. Further, this impairment correlated with self-reported anhedonic symptoms (Pizzagalli et al., 2008). These findings were recently replicated and extended by showing that reward learning was reduced in depressed patients with high levels of anhedonia symptoms, compared to patients with low levels. Furthermore, reduced reward learning at entry increased the odds for the depression diagnosis to persist after 8 weeks of treatment (Vrieze et al., 2013). Recently, impaired reward learning was even reported in patients with remitted depression (Pechtel et al., 2013). In line with these findings, a recent study using the EEfRT task reported that depressed patients were less able to effectively use information about magnitude and probability of reward to guide their choice behavior (Treadway et al., 2012a).

Related to this, several studies have used probabilistic learning tasks that differentiate between reward and punishment learning, i.e., learning “by carrot or by stick”, and have shown impairments in reinforcement learning in patients suffering from depression and schizophrenia. Patients suffering from schizophrenia have been consistently found to exhibit deficits in reward-driven learning (Waltz et al., 2007, 2011; Strauss et al., 2011; Gold et al., 2012; Yilmaz et al., 2012; Fervaha et al., 2013a), while findings regarding punishment-driven learning are more conflicting. In most studies punishment-driven learning is seemingly intact, but a few recent studies also report impairments in punishment-driven learning (Fervaha et al., 2013a; Reinen et al., 2014).

Less data is available on depressed patients, but Chase et al. reported evidence of blunting in terms of smaller learning rates in both positive and negative learning in a group of depressed patients (Chase et al., 2010). Notably, the diagnosis group accounted for considerably less of the variance in blunting than individual differences in anhedonia, and the effect of depression on blunting was very small if anhedonia was factored out.

Interestingly, human studies have shown that even without conscious processing of contextual cues, the brain can learn their reward value and use them to provide a bias on decision making. In a subliminal instrumental conditioning task, where the cues predicting monetary reward or punishment were subliminal, participants still developed a propensity to choose cues associated with monetary rewards relative to punishments (Pessiglione et al., 2008). These findings support the notion that reward and punishment also affect our behavior outside of our awareness, thereby underscoring the problems inherent in relying (only) on self-report measures. This type of paradigm has not been applied to patient groups yet, but represents a promising way to investigate possible impairments in implicit learning.

In general, isolating reward learning from motivational processes and hedonic impact is challenging. Although the presented tasks focus on reward learning, aspects of wanting and liking may interact and affect findings. For example, in *the probabilistic reward task* adopted by Pizzagalli et al. one of the main outcomes is the development of a bias toward the most frequently rewarding stimulus. Although development of this bias represents an ability to optimize behavior based on reinforcement history, the task does not allow a complete disentanglement of wanting, liking and learning. Development of this bias is likely to be influenced by reward wanting, and since development of a positive response bias also reflects an ability to value high reward more than low reward, reward liking may interact.

#### Neuroimaging measures of reward learning

In recent years, there has been a growing interest in studying impairments in reinforcement learning and corresponding brain activity in patients suffering from depression and schizophrenia. Some of these studies have investigated brain responses to expectation and receipt of reward and punishment using Pavlovian (i.e., passive) and instrumental (i.e., active) learning paradigms. Related to this are also findings from the mentioned MID task (Knutson et al., 2000), which can be used to examine responses to reward receipt (i.e., liking), but may also inform us on associative learning by looking at neural responses during reward expectation and reward receipt.

Several studies have applied these paradigms to patients suffering from schizophrenia to examine whether abnormalities in reward expectation and prediction error signals (i.e., differences between expected and actual outcome) could underlie negative symptoms by disrupting learning and blunting the salience of rewarding events. Overall, studies have revealed blunted ventral striatal responses to cues predicting reward, which has been associated with severity of negative symptoms in some studies (Juckel et al., 2006a,b; Simon et al., 2010; Dowd and Barch, 2012). Similarly, there is evidence of blunted striatal activity in response to prediction errors (i.e., responses that do not match expectations) or reward receipt (Schlagenhauf et al., 2009; Waltz et al., 2009; Koch et al., 2010; Gradin et al., 2011), although some studies have reported almost intact neural responses (Simon et al., 2010; Waltz et al., 2010; Dowd and Barch, 2012). This inconsistency of findings may be partly explained by the fact that schizophrenia patients are a heterogeneous group. Importantly, several of these studies found an association between reduced striatal responses to reward receipt and increased negative or depressive symptoms (Waltz et al., 2009, 2010; Simon et al., 2010).

Findings from studies of depressed patients also report blunted striatal responses to reward learning, although less data is available. Using a Pavlovian learning task during fMRI, Kumar et al., reported blunted responses to reward learning signals in depressed patients in regions including ventral striatum and midbrain (Kumar et al., 2008). Similar findings were reported using an instrumental learning task. Compared to controls, depressed patients had reduced activity associated with prediction errors in the striatum and midbrain, and the extent of signal reduction correlated with increased (self-reported) anhedonia severity (Gradin et al., 2011). None of these studies reported behavioral differences between depressed patients and controls (i.e., self-reported pleasure from the reward, accuracy).

In contrast to this, Steele et al. reported a blunted response in depressed patients in both behavioral and neural responses (measured with fMRI) to feedback information during a gambling task (Steele et al., 2007). Control participants responded to losses by an increase in reaction time and activity of the anterior cingulate cortex, while patients did not increase their reaction times or activity in the anterior cingulate cortex. Similarly, controls responded to wins by a reduction in reaction times and activity in the ventral striatum, while patients showed none of these effects. Further, self-reported anhedonia correlated with reaction time adjustment, with increases in anhedonia being associated with smaller reaction time effects.

These findings are in line with findings from e.g., Chase et al. who also found support for blunting both in terms of positive and negative feedback (Chase et al., 2010). Further, measures of self-reported anhedonia seem to be modulating the magnitude of these parameters with increasing anhedonia being associated with reduced effects.

The study of subliminal instrumental conditioning by Pessiglione et al. also allowed for analysis of corresponding patterns of brain activity using fMRI data (Pessiglione et al., 2008). During conditioning, both cue values and prediction errors correlated with activity in the ventral striatum. Hence, activity patterns were similar to those found in studies using paradigms where contextual cues are consciously perceived (Pagnoni et al., 2002; O’Doherty et al., 2004; Pessiglione et al., 2006).

#### Summary

Taken together, the bulk of the evidence suggests that anhedonia is associated with a blunted or attenuated ability to learn to respond to feedback information, i.e., problems with learning reinforcement to alter behavior in patients suffering from depression and schizophrenia. This is particularly evident in terms of reward-driven learning, while findings regarding punishment-driven learning are mixed in patients suffering from schizophrenia. The attenuated ability to learn from feedback information is supported in neuroimaging studies by revealing blunted ventral striatal responses during learning in patients suffering from schizophrenia and depression, which in some cases was associated with severity of self-reported anhedonia symptoms. It is also notable that instrumental learning outside conscious awareness produces similar activity in brain reward networks to what has been reported in conscious instrumental conditioning studies.

### Reconceptualizing anhedonia in psychiatric disorders

Based on the presented evidence, it is difficult to maintain a view of anhedonia as a unitary process, which only manifests itself in the reduced ability to experience subjective pleasure. Instead, the available data strongly suggests that anhedonia should be redefined to reflect the heterogeneous nature of hedonic processing across disorders and individuals. We therefore propose to reconceptualize anhedonia as the breakdown or unbalancing of any or all of the complex psychological processes involved in reward processing as it unfolds over time in the pleasure cycle (Figure 1). In the normal brain, wanting, liking and learning processes are balanced over time (Figure 2). However, impairments in each of the subcomponents of reward can lead to specific symptoms (or subtypes) of anhedonia that are associated with specific imbalances between wanting, liking and learning processes in the brain. In order to disentangle the engagement of the various reward components, behavioral or physiological measures are needed to complement self-report measures, which will help in terms of quantifying core “liking”, “wanting” and “learning” components, as well as their explicit counterparts.

The currently available data does not support the notion that all components of hedonic processing are compromised at the same time in various psychiatric disorders. Instead, perhaps surprisingly, some aspects of *conscious liking*—which is what most people intuitively associate with pleasure—can be seemingly intact in the psychiatric disorders traditionally associated with anhedonia, including depression and schizophrenia. In contrast, *wanting* and *learning* components are more easily compromised (see Figure 2). Many studies of patients suffering from depression and schizophrenia show deficits in these domains, for example in terms of reduced willingness to work for a reward, and reduced ability to learn from reward and punishment, while some aspects of liking can be seemingly intact (as reviewed in sections Impairments in liking, Impairments in wanting and Impairments in reward learning).

This raises the interesting question that if liking is in fact intact (as shown in experimental taste-reactivity investigations), why do patients suffering from depression and schizophrenia subjectively report this symptom in clinical inventories and interviews? One possibility is that core “liking” reactions remain intact, yet patients no longer *cognitively* value them as they did before (Dichter et al., 2010; Berridge and Kringelbach, 2011). This interpretation is supported by data showing that while online measures of hedonic impact are intact, patients suffering from depression and schizophrenia report reduced enjoyment when asked to rate future, past or hypothetical experience (McFarland and Klein, 2009; Watson and Naragon-Gainey, 2010; Strauss and Gold, 2012), which is standard in most clinical interviews assessing anhedonia.

At the same time, this interpretation has to be seen in the light of standard clinical examinations of patients, where depression with anhedonia is associated with direct behavioral characteristics implying a problem that is not only related to cognitive evaluations of past and future. For example, clinicians often report fewer facial expressions, less smiling, less reactivity to stimuli and other types of symptoms that could reflect diminished “liking”. This disagreement between clinical observations and findings from studies applying taste-reactivity paradigms in humans stresses the need to consider methodological aspects. The seemingly intact “liking” reactions to pleasurable solutions and odors are based on self-report measures, and it is possible that behavioral or physiological measures will inform us differently.

Another possibility is that core “liking” reactions are intact in some subtypes of anhedonia, but suppressed in other types, and correspondingly with the cognitive evaluations. One of the main reasons for our reconceptualization is to stress the notion that anhedonia is not a unitary process, but is instead a complex psychological process which consists of several subcomponents that can occur on different levels of conscious awareness and control (similar to reward). As reviewed here, deficits in each of the reward components, and corresponding imbalance between components, can lead to different expressions, or subtypes of anhedonia.

In future, it may be more useful to define separate subtypes of anhedonia, reflecting impairments in the specific reward components. In line with this reasoning, Treadway and Zald have suggested to differentiate between motivational, consummatory and decisional anhedonia (Treadway and Zald, 2011). It may even be more useful to replace the term anhedonia with more functional terms such as diminished (or elevated) reward wanting, and diminished (or elevated) reward liking. Although anhedonia has traditionally been associated with diminished responses, our proposed framework acknowledges that both too much and too little activity in specific parts of the pleasure system can lead to pathological changes. This is for example illustrated in the excessive wanting for drugs in drug addiction or in disorders with hypersexuality.

Related to this is also the well-documented negative response bias in (at least) depressed patients (Leppanen, 2006), which may account for some of the discrepancy between what patients report in here-and-now situations, and how they cognitively value past and future events. The lateral habenula is known to be a key structure in mediating the response to emotionally negative states (Hikosaka et al., 2008; Hikosaka, 2010), as well as the balance of activity between the amygdala and nucleus accumbens (Willner et al., 2013). Increased activity in the lateral habenula (induced e.g., by stress) can lead to an increase in the salience of aversive stimuli and a decrease in the saliency of appetitive stimuli, and as such offers a plausible neurobiological substrate for the negative information-processing bias seen in e.g., depressed patients (Disner et al., 2011; Willner et al., 2013). Dysfunctions of this limbic-striatal relay nucleus have been implicated in depression and schizophrenia (Hikosaka et al., 2008), and recently beneficial effects were reported in a treatment-resistant depressed patient receiving deep brain stimulation in this target (Sartorius et al., 2010).

Overall, more studies are needed before we can make conclusive statements regarding the role of wanting, liking, and learning processes in anhedonia in psychiatric disorders. In particular, development of valid behavioral or physiological measures of hedonic impact is needed before we can make any conclusive statements regarding the role and nature of liking processes in anhedonia. As already stressed, the current finding that here-and-now measures of hedonic reactivity can be intact (in depressed and schizophrenic patients) is based on self-report alone. Future studies applying behavioral or physiological measures of “liking” in studies of anhedonia might inform us differently.

In addition to depression and schizophrenia, which have traditionally been associated with anhedonia, there has been a growing interest in the role of anhedonia across disorders, in particular addictive disorders, including gambling disorder (Ahmed and Koob, 1998; Markou et al., 1998; Koob and Le Moal, 2001; Volkow et al., 2002; Rømer Thomsen et al., 2009; Hatzigiakoumis et al., 2011), eating disorders (Davis and Woodside, 2002; Jiang et al., 2010; Keranen et al., 2010; Tchanturia et al., 2012), and Parkinson’s disease (Isella et al., 2003; Loas and Krystkowiak, 2010; Santiago et al., 2010; Loas et al., 2012).

In addictive disorders, motivational processes are more pertinent than liking *per se*, and overall addictions represent a clear example of how wanting can be dissociated from liking. In contrast to depression, drug addiction is characterized by an *excess* of drug wanting, which is rarely accompanied by the expected feeling of pleasure in pathological cases (Figure 2). Further, the excessive and never-ending chase of the reward of choice leaves little room for the pursuit of other pleasures. In other words, drug craving is expressive of an unhealthy form of wanting that pushes aside goal-directed behavior toward other pleasurable activities, as described in the influential incentive-sensitization theory of drug addiction (Robinson and Berridge, 1993; Robinson et al., 2013). Similar mechanisms are likely to be at play in behavioral addiction, such as gambling disorder, which is also characterized by an excess of wanting, that is rarely matched by the expected feeling of liking (Rømer Thomsen et al., 2014). Until recently, gambling disorder was classified as an impulse control disorder (American Psychiatric Association, 1994). However, due to a large overlap with drug addiction in terms of clinical symptoms and underlying neurobiology, there has been a growing agreement to view gambling disorder as a behavioral addiction (Russell, 1996; Gold et al., 2008; Potenza, 2008; McCabe et al., 2009; Smoski et al., 2009; Frascella et al., 2010), which has been acknowledged in the DSM-5 (American Psychiatric Association, 2013).

Another class of psychiatric disorders, eating disorders, could benefit from a reconceptualization of anhedonia. Berridge et al. suggested that patients suffering from some forms of eating disorders can be characterized as having normal levels of wanting, but low levels of liking of food (Berridge et al., 2010). In other types of eating disorders this pattern is reversed. Binge eating, for example, may be characterized by an excess of wanting, which is rarely followed by the expected feeling of pleasure, similar to drug and behavioral addiction. For some patients (at the severe end of the continuum) their eating disorder may in fact resemble addiction, and should perhaps be termed food addiction. However, this group of patients would appear to be relatively small (Berridge et al., 2010).

Additional support for the important role of motivational processes and underlying mesolimbic dopamine systems comes from the study of Parkinson’s Disease. While brief administration of dopamine agonists showed improved willingness to work for a reward in healthy controls (Wardle et al., 2011), long-term treatment with dopamine agonists in Parkinson’s patients can cause compulsive behavior, such as pathological gambling activity or hyper-sexuality in some patients (Weintraub et al., 2006; Voon et al., 2009, 2011). As suggested by our reconceptualization of anhedonia, it would appear that both too much and too little activity in specific components can lead to pathological changes (Kringelbach et al., 2012; Robinson et al., 2013). It would be of considerable interest to carry out studies of anhedonia in this patient group. Interestingly, it was recently shown that apathy in some Parkinson’s patients is related to goal-directed behavior and anticipatory, but not consummatory, anhedonia (Jordan et al., 2013), supporting our proposed reconceptualization of anhedonia.

Taken together, the available data suggests that anhedonia is heterogeneous across disorders. Considerable progress can be expected when a deeper understanding of the interplay and balance between each of the underlying reward components in disorders is gained. Improving our understanding of the neurobiological underpinnings of specific behavioral disruptions such as anhedonia is crucial because it will facilitate treatment of disorders that include such symptoms (Der-Avakian and Markou, 2012). The development of objective behavioral measures of e.g., “wanting” processes can facilitate this process and help elucidate the neurobiology of impairments in the ability to *seek* pleasure. This work has already begun, for example with the EEfRT. However, patient groups have yet to be extensively tested using behavioral measures of *wanting, liking*, and *learning* in combination with neuroimaging, which leaves much scope for better characterization of the various imbalances in the human pleasure networks.

## Implications for diagnosis and treatment of anhedonia

Our proposal of anhedonia as impairments in specific reward components and corresponding unbalancing of pleasure networks both broadens and strengthens the use of anhedonia in providing useful diagnostic markers for mental illness. As such it offers a number of potential test instruments that could be more reliable and specific than the existing self-report questionnaires for anhedonia. These tests may offer greater specificity in diagnosing anhedonia in many heterogeneous psychological disorders, where symptoms may be expressed differently across people, or even differently across time within the same individual (Nelson et al., 2009).

Take depression as an example. In the DSM-5, anhedonia is described as “decreased interest and pleasure in most activities most of the day” (American Psychiatric Association, 2013), thereby collapsing wanting and liking. This is in contrast to the large literature suggesting that these processes are in fact dissociable. Although patients suffering from depression often report reduced enjoyment on a cognitive level (measured in clinical interviews and self-report inventories), there is evidence that not all patients have similar impairments in core “liking” reactions. At the same time there is increasing evidence of impairments in reward motivation and reward learning in depressed patients. Considering that there is compelling evidence that wanting, liking and learning processes are not subserved by the exact same networks in the brain (e.g., mesolimbic dopamine is more involved in wanting than liking), potential future medical (and psychological) treatment could be informed and improved by a better characterization of the specific reward-related deficits in individual patients. As a start, self-report measures of enjoyment could usefully be complemented with behavioral measures of motivation and learning.

Animal behavioral paradigms have been developed that measure specific components of reward processing, and there has been recent progress in developing translational tools for use in humans. Hopefully these measures will continue to be developed and applied to relevant patient groups, and in time help us obtain a fuller picture of anhedonia, and consequently help improve treatment options. In particular, tests that tap into the unconscious components of this processing can be very useful. For example, wanting processes that occur outside of awareness are important for addiction, as outlined by the incentive-sensitization theory (Robinson et al., 2013). This acknowledgment of unconscious mechanisms has implications for treatment. e.g., cognitive behavioral therapy is important in terms of targeting conscious craving mechanisms in addiction (Potenza et al., 2011), but although it reduces some layers of responsiveness to drug-cues, unconscious layers are likely to persist (Robinson et al., 2013). In contrast, mindfulness-based interventions can potentially target unconscious “wanting” mechanisms by increasing awareness of bodily and emotional signals (Garland et al., 2014). Preliminary findings show that these treatments reduce consumption of several substances and is associated with a reduction in craving in substance users although more randomized controlled trials are warranted (Chiesa and Serretti, 2014).

Given the identification of the importance of the motivational component of anhedonia, and given the well-documented role of dopamine in incentive salience processing, it is important to acknowledge the role of dopamine in the study of anhedonia (and disorders characterized by anhedonic symptoms such as depression). Improving current treatments for e.g., depression may well be aided by a conceptual shift towards focusing on anhedonia and the role of dopamine in the interaction with serotonin. Evidence for such a shift comes from a number of sources including convergent findings from neuroimaging, post-mortem, behavioral and pharmacological studies pointing to a reduced dopamine function in depression (Kumar et al., 2008). This should also be seen in the context of the emerging evidence that treatments solely targeting serotonergic noradrenergic systems have limited efficacy, e.g., as shown in meta-analyzes of antidepressant efficacy compared to placebo (Kirsch et al., 2008). The findings reviewed here point to an important role of mesolimbic dopamine and opioids in anhedonia symptoms, and are in line with recent proposals to target these neurotransmitters more directly in depressed, or anhedonic, patients (Kumar et al., 2008; Treadway and Zald, 2011; Soskin et al., 2013).

## Conclusion

This review has discussed the emerging evidence for the functional neuroanatomy of pleasure and shown how the specific breakdown of any or all of the underlying components of wanting, liking, and learning can lead to a malfunctioning pleasure system. This in turn can be conceptualized as anhedonia, the lack of ability to *experience*, *pursue*, and/or *learn about* pleasure. We discussed the heterogeneity of anhedonia across psychiatric disorders and specifically pointed out the dissociation between wanting, liking, and learning components. We reviewed the behavioral and neuroimaging studies of anhedonia as the reduced ability to experience, pursue, and learn from pleasure, and stressed the importance of including their nonconscious counterparts. This pointed to a pertinent role of both wanting, liking, and learning components, which is in contrast to the traditional view of anhedonia as (only) reduced subjective liking. In fact, the evidence suggested that here-and-now measures of pleasure liking are seemingly intact in patients suffering from depression and schizophrenia (although this may be due to methodological challenges). In contrast, wanting and learning components are more easily compromised in these patients, for example in terms of reduced willingness to work for a reward, and reduced ability to learn from reward and punishment. Related to this, evidence from animal studies supports the notion that the capacity for “liking” reactions is rather robust in the brain, by showing that only one of the hedonic hotspots in the posterior ventral pallidum is necessary for “liking” (Cromwell and Berridge, 1993; Smith et al., 2010).

The findings reviewed here should, however, be seen in the context of a number of limitations or caveats. First of all, the reported findings of normal levels of pleasure liking in here-and-now measurements in depressed and schizophrenic patients are based on self-report. Development of valid behavioral measures of “liking” reactions that can be applied in human studies are needed before we can make conclusive claims. In contrast, behavioral measures of “wanting” and “learning” mechanisms have been succesfully translated from animal to human studies. Some of these measures have started to be applied in relevant patient groups and offer intriuging new insights on the reduced ability to seek and learn about pleasure. However, patient groups have yet to be extensively tested using behavioral measures of *wanting, liking*, and *learning* in combination with neuroimaging, which leaves much scope for a better characterization of the underlying neurobiology. New imaging techniques, in particular magnetoencephalography (MEG), which offers a unique combination of high temporal and spatial resolution, represent promising new tools to capture and tease apart the rapid emotional processes likely to occur outside of our awareness.

Another important caveat is that so far the majority of human studies of the brain regions involved in anhedonia have been correlative in nature, thereby limiting our knowledge of the underlying brain circuitries. We need a better understanding of which brain regions are sufficient and necessary for rebalancing pleasure networks in neuropsychiatric disorders. Such knowledge is difficult to obtain from human studies, although new information is trickling in from studies using causal methods such as psychopharmacological methods with e.g., conditioned place preferences (Mayo et al., 2013; Mayo and De Wit, 2015) as well as more direct brain manipulations such as transcranial magnetic stimulation, transcranial direct current stimulation and deep brain stimulation (Kringelbach et al., 2007, 2011; Lozano and Lipsman, 2013).

In addition, computational neuroscience may help generate new information. The progress in using diffusion tensor imaging methods to track changes in brain connectivity in neuropsychiatric disorders together with the high temporal and spatial resolution of MEG will allow us to make computational models that can accurately predict the functional consequences of structural abnormalities. In time, this new understanding may lead to more precise diagnoses and treatments of anhedonia (Deco and Kringelbach, 2014).

## Conflict of interest statement

The authors declare that the research was conducted in the absence of any commercial or financial relationships that could be construed as a potential conflict of interest.
